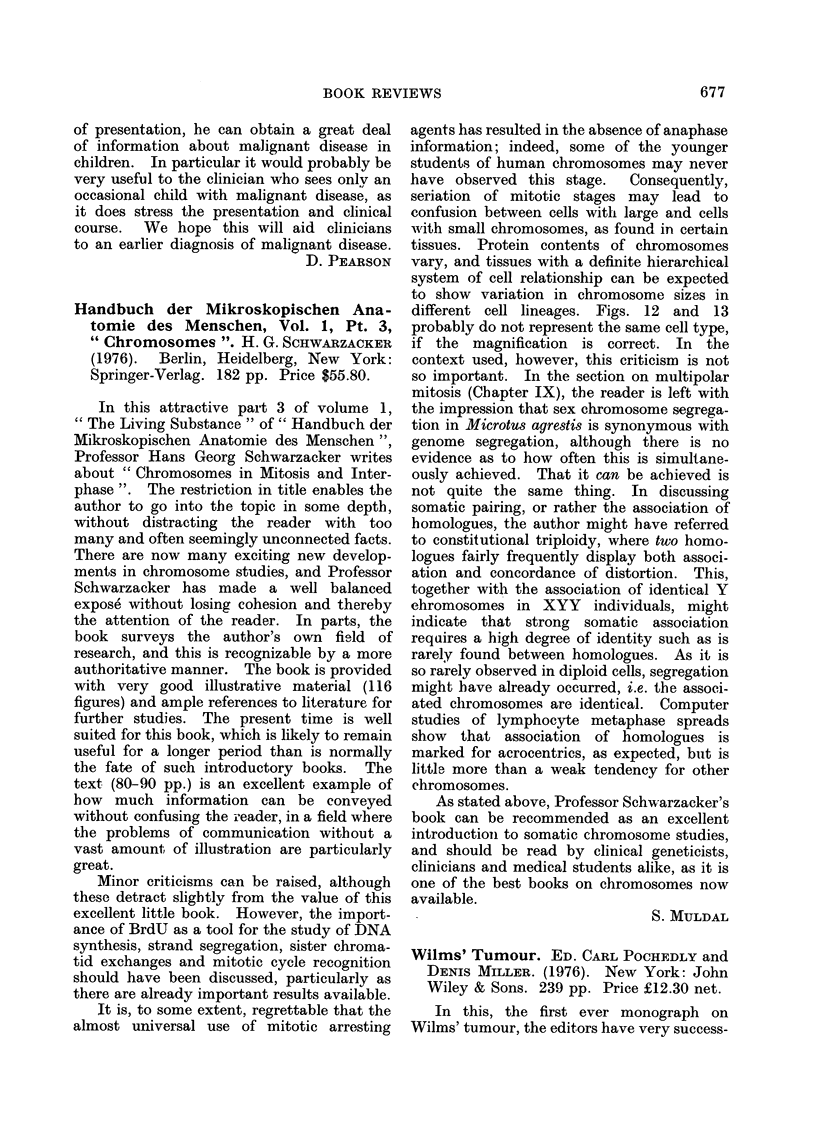# Handbuch der Mikroskopischen Anatomie des Menschen, Vol. 1, Pt. 3, “Chromosomes”

**Published:** 1976-12

**Authors:** S. Muldal


**Handbuch der Mikroskopischen Anatomie des Menschen, Vol. 1, Pt. 3, " Chromosomes ".** H. G. SCHWARZACKER (1976). Berlin, Heidelberg, New York: Springer-Verlag. 182 pp. Price $55.80.

In this attractive part 3 of volume 1, " The Living Substance " of " Handbuch der Mikroskopischen Anatomie des Menschen ", Professor Hans Georg Schwarzacker writes about " Chromosomes in Mitosis and Interphase ". The restriction in title enables the author to go into the topic in some depth, without distracting the reader with too many and often seemingly unconnected facts. There are now many exciting new developments in chromosome studies, and Professor Schwarzacker has made a well balanced exposé without losing cohesion and thereby the attention of the reader. In parts, the book surveys the author's own field of research, and this is recognizable by a more authoritative manner. The book is provided with very good illustrative material (116 figures) and ample references to literature for further studies. The present time is well suited for this book, which is likely to remain useful for a longer period than is normally the fate of such introductory books. The text (80–90 pp.) is an excellent example of how much information can be conveyed without confusing the reader, in a field where the problems of communication without a vast amount of illustration are particularly great.

Minor criticisms can be raised, although these detract slightly from the value of this excellent little book. However, the importance of BrdU as a tool for the study of DNA synthesis, strand segregation, sister chromatid exchanges and mitotic cycle recognition should have been discussed, particularly as there are already important results available.

It is, to some extent, regrettable that the almost universal use of mitotic arresting agents has resulted in the absence of anaphase information; indeed, some of the younger students of human chromosomes may never have observed this stage. Consequently, seriation of mitotic stages may lead to confusion between cells with large and cells with small chromosomes, as found in certain tissues. Protein contents of chromosomes vary, and tissues with a definite hierarchical system of cell relationship can be expected to show variation in chromosome sizes in different cell lineages. Figs. 12 and 13 probably do not represent the same cell type, if the magnification is correct. In the context used, however, this criticism is not so important. In the section on multipolar mitosis (Chapter IX), the reader is left with the impression that sex chromosome segregation in *Microtus agrestis* is synonymous with genome segregation, although there is no evidence as to how often this is simultaneously achieved. That it *can* be achieved is not quite the same thing. In discussing somatic pairing, or rather the association of homologues, the author might have referred to constitutional triploidy, where *two* homologues fairly frequently display both association and concordance of distortion. This, together with the association of identical Y chromosomes in XYY individuals, might indicate that strong somatic association requires a high degree of identity such as is rarely found between homologues. As it is so rarely observed in diploid cells, segregation might have already occurred, *i.e.* the associated chromosomes are identical. Computer studies of lymphocyte metaphase spreads show that association of homologues is marked for acrocentrics, as expected, but is little more than a weak tendency for other chromosomes.

As stated above, Professor Schwarzacker's book can be recommended as an excellent introduction to somatic chromosome studies, and should be read by clinical geneticists, clinicians and medical students alike, as it is one of the best books on chromosomes now available.

S. MULDAL